# Research Trends and Evolution in Radiogenomics (2005-2023): Bibliometric Analysis

**DOI:** 10.2196/51347

**Published:** 2024-07-09

**Authors:** Meng Wang, Yun Peng, Ya Wang, Dehong Luo

**Affiliations:** 1 Department of Radiology National Cancer Center/National Clinical Research Center for Cancer/Cancer Hospital & Shenzhen Hospital Chinese Academy of Medical Sciences and Peking Union Medical College Shenzhen China; 2 Department of Radiology The Second Affiliated Hospital, Jiangxi Medical College, Nanchang University Nanchang China

**Keywords:** bibliometric, radiogenomics, multiomics, genomics, radiomics

## Abstract

**Background:**

Radiogenomics is an emerging technology that integrates genomics and medical image–based radiomics, which is considered a promising approach toward achieving precision medicine.

**Objective:**

The aim of this study was to quantitatively analyze the research status, dynamic trends, and evolutionary trajectory in the radiogenomics field using bibliometric methods.

**Methods:**

The relevant literature published up to 2023 was retrieved from the Web of Science Core Collection. Excel was used to analyze the annual publication trend. VOSviewer was used for constructing the keywords co-occurrence network and the collaboration networks among countries and institutions. CiteSpace was used for citation keywords burst analysis and visualizing the references timeline.

**Results:**

A total of 3237 papers were included and exported in plain-text format. The annual number of publications showed an increasing annual trend. China and the United States have published the most papers in this field, with the highest number of citations in the United States and the highest average number per item in the Netherlands. Keywords burst analysis revealed that several keywords, including “big data,” “magnetic resonance spectroscopy,” “renal cell carcinoma,” “stage,” and “temozolomide,” experienced a citation burst in recent years. The timeline views demonstrated that the references can be categorized into 8 clusters: lower-grade glioma, lung cancer histology, lung adenocarcinoma, breast cancer, radiation-induced lung injury, epidermal growth factor receptor mutation, late radiotherapy toxicity, and artificial intelligence.

**Conclusions:**

The field of radiogenomics is attracting increasing attention from researchers worldwide, with the United States and the Netherlands being the most influential countries. Exploration of artificial intelligence methods based on big data to predict the response of tumors to various treatment methods represents a hot spot research topic in this field at present.

## Introduction

Radiogenomics is an emerging technology that combines radiomics and genomics, with the ultimate goal of improving prognosis and outcomes [1]. Radiogenomics can be used to investigate the relationship between imaging features and gene mutations and expression patterns [2-4]. Unlike traditional gene sequencing methods, which are associated with inherent drawbacks such as invasive and high-cost procedures, radigenomics provides a noninvasive, convenient, and cost-effective method by using quantitative imaging parameters extracted from the entire lesions [5,6]. Many scholars have demonstrated that radiogenomics may predict the pathologic type, prognosis, and outcome of cancers, including lung cancer and liver cancer, based on pretreatment multimodal imaging (computed tomography [CT] or magnetic resonance imaging [MRI]) [7-10]. This technology has also been proposed as a useful biomarker for nontumor diseases, such as in the diagnosis, classification, and prognostic assessment of coronary heart disease [11].

Radiogenomics is an important potential tool for precision medicine. Some review articles summarized the routine process of radiogenomics and its various applications in the management of disease [12,13]. However, these reviews generally focused on presenting the research directions rather than analyzing the dynamic changes in the field, only highlighting the process and application status of radiogenomics. Bibliometrics can be used to quantitatively analyze the countries, institutions, authors, keywords, and other information related to the entire body of literature in a specific field. This approach can also help to visually display the dynamic progress in the field through network mapping [14]. Therefore, the aim of this study was to summarize the research status and dynamic changes of research hot spots in radiogenomics over time using bibliometric methods, thus providing a comprehensive understanding of the emerging trends in this field.

## Methods

### Bibliometric Data Acquisition

The published literature on radiogenomics was retrieved from the Web of Science Core Collection (WoSCC), which is the most widely used database in bibliometric analysis, on March 1, 2024 [15]. The initial search phase showed that the first relevant article in this field was published in 2005; hence, we restricted the publication time period to 2005-2023 [16]. The search string was as follows: “(TS=(Radiogenomics) OR TS=(Radiomics AND genomics) OR TS=((Radiomics) AND (gene* OR DNA OR RNA OR expression OR mutation OR molecular subtype))) AND FPY=(2005-2023).”

The literature retrieval and refining processes were carried out by one author (YW), while the other authors supervised the whole process. A total of 3669 documents were retrieved. The refine function of the WoSCC website was used to screen the language and document type of the retrieved documents sequentially. The exclusion criteria were as follows: (1) document type is not a research or review article (eg, proceeding paper, meeting abstract, or editorial) and (2) articles written in languages other than English (eg, Japanese or Chinese). There were 420 papers excluded due to an inappropriate document type and 12 papers excluded due to language; thus, 3237 papers remained for analysis, which were exported in plain-text format. The information on corresponding countries, institutions, authors, and journals obtained from the WoSCC were recorded. The original data of the retrieved articles are provided in Multimedia Appendix 1.

### Data Analysis and Visualization

After detecting duplicate documents using CiteSpace (version 6.3.R1), Excel 2016, CiteSpace, and VOSviewer (version 1.6.19.0) were used to perform the bibliometrics analysis. Excel was used to analyze the annual publications, whereas VOSviewer was used to generate visualization networks of keywords co-occurrence and of collaboration among countries and institutions. The networks were constructed with keywords, countries, and institutions as nodes, respectively. The thickness of the line connecting nodes indicates the strength of the association. The betweenness centrality (BC) parameter was used to assess the importance of each node in the network; a higher BC value signifies greater importance in the network [17].

CiteSpace was used to determine the citation burst of keywords and to map the timeline of references by keywords. Burst detection can detect the citation burst of a specific keyword (or document) within a certain field, at least within a short period of time during a given time frame [18]. Keywords with a burst of occurrences are indicators of hot spot research topics in a field. According to the instructions of CiteSpace [19], the timeline of reference clusters is an analysis based on references for the exported literature. CiteSpace can extract noun phrases from the titles, keyword lists, or abstracts of articles that cited the particular cluster. The automatically selected labels will be displayed and the clusters are numbered in descending order of the cluster size, starting from the largest as cluster 0, the second largest as cluster 1, and so on. In this way, the network characterizes the development of the field over time, showing the most important footprints of the related research activities.

Certain data, including the number of publications, impact factor, h-index, and average per item of journals, countries, and authors, were retrieved from the WoSCC website. The h-index and average per item were used in the country, institution, and author analyses. The h-index was introduced by Hirsch [20] in 2005 and is commonly used as a scientific contribution metric corresponding to the number of times a paper is cited. The average per item is calculated by dividing the total number of citations by the number of publications, resulting in the average number of citations per publication.

### Synonym Substitution of Keywords

**Figure 1:**
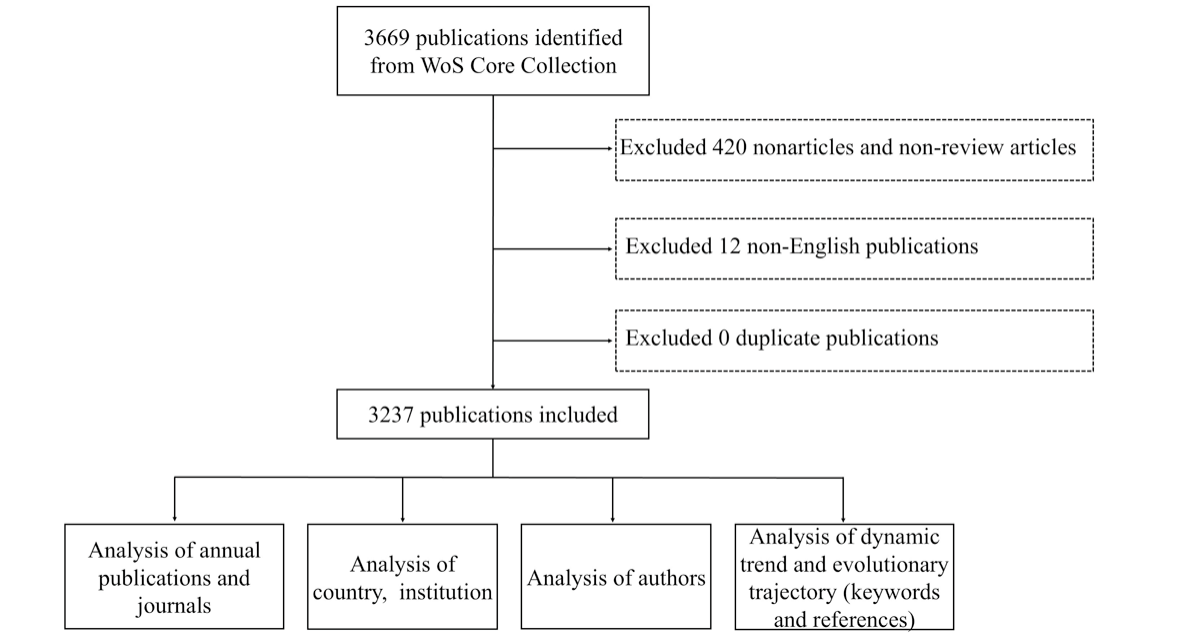
Workflow of the analytical procedures. WoS: Web of Science.

The keywords with the same meaning were merged by synonym substitution. For example, terms such as “computed tomography,” “computed tomography (ct),” “computed-tomography,” “ct,” and “ct images” were uniformly labeled as “CT.” The full list of keyword synonyms is provided in Multimedia Appendix 2. Figure 1 shows a workflow of the analytical procedures.

## Results

### Annual Publications Trend

**Figure 2:**
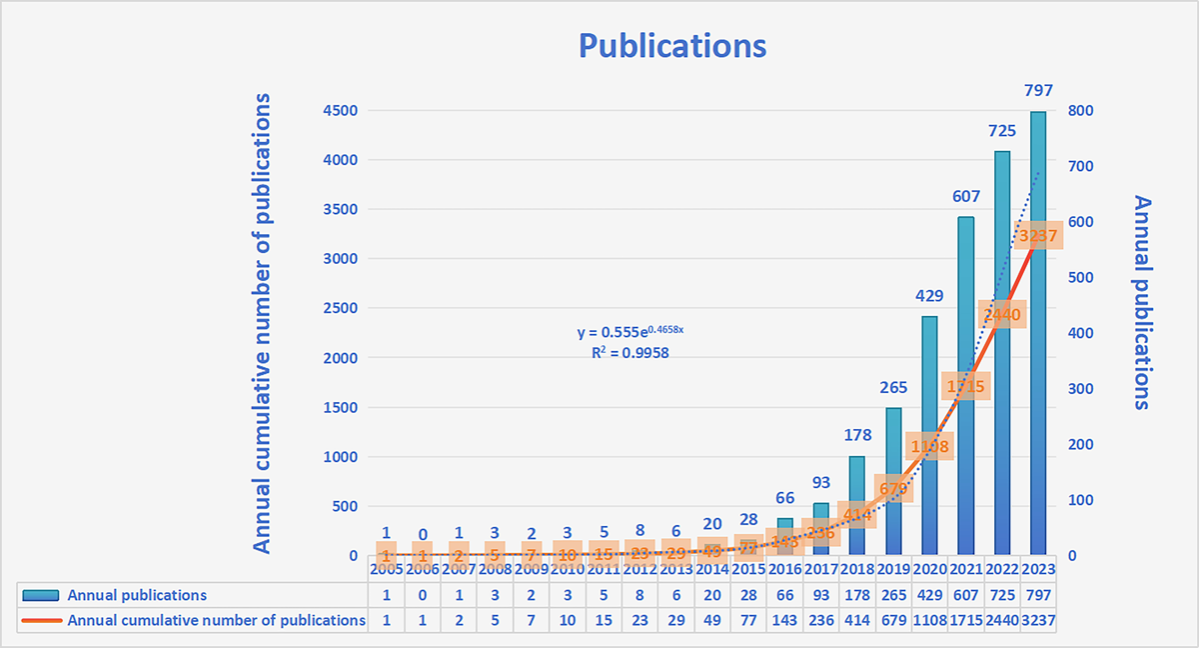
Global trend of publications on radiogenomics from 2005 to 2023.

A total of 3237 papers were included for the final analysis. No duplicate article was found. The results indicated a clear upward trend in research on radiogenomics since 2013 (Figure 2).

### Journals

Table 1 presents the top 15 journals with the highest number of publications on radiogenomics. *Frontiers in Oncology, Cancers, and European Radiology* were the top three journals publishing in this field with 284, 196, and 135 papers, respectively.

**Table 1:** Top 15 journals publishing in the field of radiogenomics.

Rank	Journal	Articles, n	IF^a^ (2022)
1	Frontiers in Oncology	284	4.7003
2	Cancers	196	5.5999
3	European Radiology	135	5.9003
4	Scientific Reports	103	4.6
5	Diagnostics	73	3.5999
6	Medical Physics	63	3.8001
7	Journal of Magnetic Resonance Imaging	60	4.3997
8	European Journal of Radiology	57	3.3
9	Academic Radiology	50	4.8003
10	Physics in Medicine And Biology	49	3.5
11	British Journal of Radiology	45	2.6002
12	Radiology	41	19.7005
13	European Journal of Nuclear Medicine and Molecular Imaging	40	9.1005
14	Abdominal Radiology	34	2.4002
15	BMC Medical Imaging	33	2.7001

^a^IF: impact factor.

### Countries and Institutions

A total of 71 countries have published articles related to radiogenomics. Table 2 highlights the top 10 countries in terms of the number of publications. China ranks first with 1470 articles, followed by the United States with 891 articles and Italy with 326 articles. The United States obtained the highest citation count and the second highest average citation per item. Only three countries, the Netherlands (99.86), the United States (52.11), and Canada (51.46), have average citations per item above 50, with the average citation count for the Netherlands standing high above those of the other countries. Six countries, including the United States, England, Italy, Canada, China, and the Netherlands, had high BC values (≥0.1).

**Table 2:** Top 10 productive countries with the most publications in the field of radiogenomics.

Rank	Total link strength	BC^a^	Country	Publications, n	H-index	Times cited	Average per item
1	329	0.11	China	1470	68	25,319	17.22
2	820	0.36	United States	891	87	46,426	52.11
3	311	0.13	Italy	326	41	7327	22.48
4	353	0.06	Germany	233	39	7966	34.19
5	418	0.19	England	209	37	6185	29.59
6	328	0.1	Netherlands	161	37	16,077	99.86
7	54	0.01	South Korea	142	31	4296	30.25
8	248	0.12	Canada	140	36	7204	51.46
9	223	0.08	France	128	32	4099	32.02
10	181	0.01	Switzerland	88	23	1893	21.51

^a^BC: betweenness centrality.

**Figure 3:**
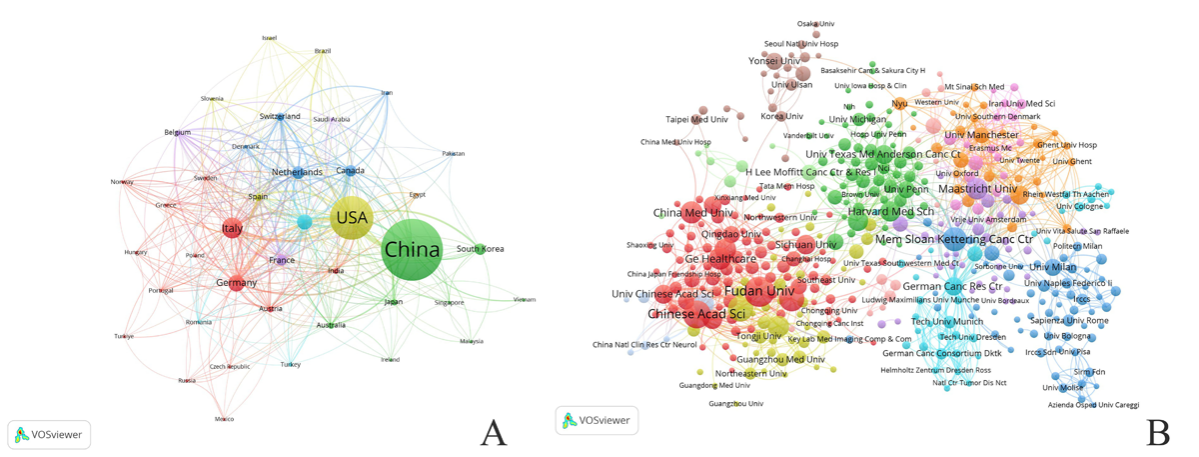
Countries (A) and institutions (B) collaboration networks in the field of radiogenomics. The size of the nodes corresponds to the number of published documents and the line width between nodes indicates the strength of coauthorship. Thicker lines indicate a higher frequency of cooperation.

Among the total 71 countries/regions that have contributed to radiogenomics research, 40 have published 5 or more documents. Figure 3A presents the visualization of the countries network. A total of 3523 institutions have contributed to radiogenomics research and 432 institutions published 5 or more documents. Figure 3B presents the visualization of the institutions network. These results show that there is relatively more cooperation between developed countries and their institutions.

### Authors

A total of 17,727 authors have contributed to radiogenomics. Table 3 presents the top 10 productive authors and the most cited authors in this field. The authors with the most publications are Philippe Lambin (33 papers), Catharine M West (32 papers), and Robyn Gillies (32 papers). Three of the top 10 productive authors, including Philippe Lambin (the Netherlands), Robyn Gillies (Australia), and Hugo Aerts (United States), also ranked in the top 3 for citations.

**Table 3:** Top 10 most productive and highly cited authors on radiogenomics.

Rank	Publications						Citations		
	Author	Articles, n	Country	H-index	Average per item	Author		Citations, n	Country
1	Philippe Lambin	33	Netherlands	21	358.06	Philippe Lambin		1573	Netherlands
2	Catharine M West	32	England	17	44.78	Robyn Gillies		1109	Australia
3	Robyn Gillies	32	Australia	24	486.13	Hugo Aerts		965	United States
4	Evis Sala	26	Italy	17	35.92	JJM Van Griethuysen		720	United States
5	Andre Dekker	25	Netherlands	12	448.4	Alex Zwanenburg		597	Germany
6	Jie Tian	25	China	14	48.04	Chintan Parmar		448	United States
7	Sarah L Kerns	25	United States	17	44.72	David N Louis		429	United States
8	Hugo Aerts	24	United States	21	526.42	Philip Kickingereder		390	Germany
9	Shaofeng Duan,	24	China	11	12.67	Yan-Qi Huang		382	China
10	Seung-Koo Lee	22	South Korea	10	23.91	Kumar Vinod		376	India

### Keywords

**Figure 4:**
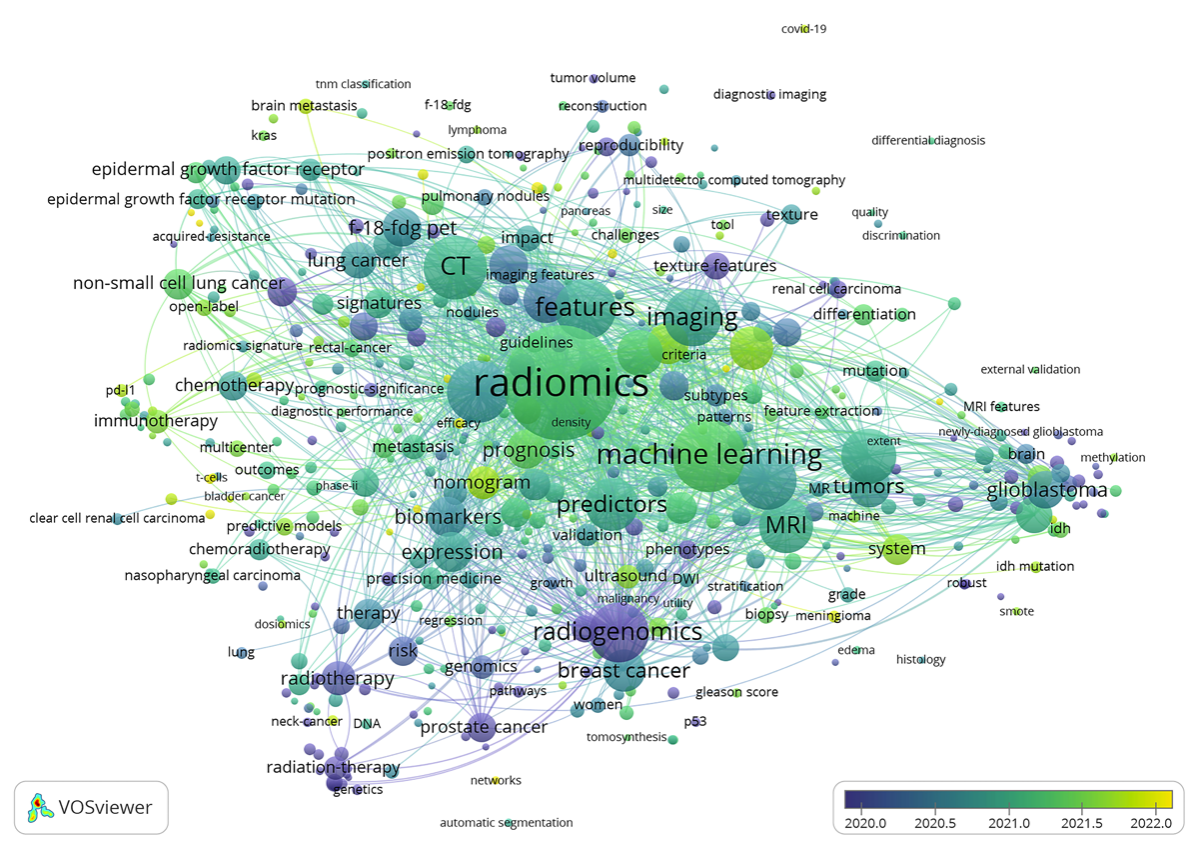
Overlay visualization map of keywords. The node size indicates the frequency of keyword occurrences and the color represents the average publication year of the identified keywords.

There were 7624 keywords identified in this study and 466 keywords appeared more than 9 times. Figure 4 presents an overlay visualization map of the co-occurring keywords.

Table 4 presents the top 30 keywords based on their occurrence frequency. Apart from “radiomics” and “radiogenomics,” the most frequent keyword was “machine learning” (n=779), followed by “CT” (n=580) and “carcinoma” (n=569).

**Table 4:** The top 30 keywords with the highest frequency in the field of radiogenomics.

Rank	Keywords	Total link strength	Frequency
1	radiomics	13,538	1918
2	machine learning	5816	779
3	CT^a^	4300	580
4	carcinoma	4250	569
5	features	4279	538
6	radiogenomics	4102	535
7	survival analysis	3984	500
8	imaging	3650	478
9	classification	3367	439
10	MRI^b^	3323	423
11	predictors	3427	416
12	diagnosis	2247	310
13	texture analysis	2489	303
14	deep learning	2076	273
15	tumors	2095	271
16	breast cancer	1958	260
17	artificial intelligence	2074	254
18	expression	1906	242
19	biomarkers	1906	240
20	heterogeneity	1902	227
21	F-18-FDG PET^c^	1874	225
22	prognosis	1670	219
23	glioblastoma	1806	212
24	gliomas	1611	198
25	lung cancer	1485	193
26	radiotherapy	1258	175
27	nomogram	1228	167
28	models	1083	152
29	recurrence	1329	152
30	system	983	144

^a^CT: computed tomography.

^b^MRI: magnetic resonance imaging.

^c^F-18 FDG PET: fludeoxyglucose F18 positron emission tomography.

### Keywords Burst and References Cluster

**Figure 5:**
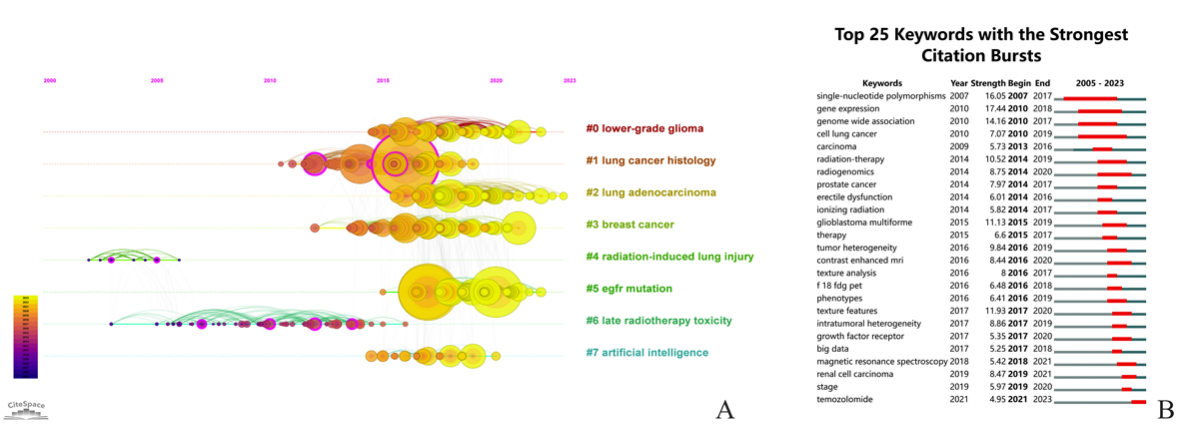
Timeline view of reference clustering analysis on radiogenomics (A) and top 25 keywords with the strongest citation bursts (B).

The top 25 keywords with the strongest citation bursts are depicted in Figure 5A. The top 3 keywords with the strongest citation bursts were “gene expression” (17.44), “single nucleotide polymorphism” (16.05), and “genome-wide association” (14.16). The keywords “big data,” “magnetic resonance spectroscopy,” “renal cell carcinoma,” “stage,” and “temozolomide” experienced a citation burst in recent years. Figure 5B illustrates the reference clusters along horizontal timelines. CiteSpace generated 8 clusters: cluster 0 for lower-grade glioma, cluster 1 for lung cancer histology, cluster 2 for lung adenocarcinoma, cluster 3 for breast cancer, cluster 4 for radiation-induced lung injury, cluster 5 for epidermal growth factor receptor (EGFR) mutation, cluster 6 for late radiotherapy toxicity, and cluster 7 for artificial intelligence.

Based on this comprehensive analysis, the time frame from 2005 to 2023 can be artificially segmented into distinct phases based on the evolution of hot topics in the field. The first phase is approximately from 2005 to 2010, represented by the keywords “radiation-induced lung injury,” “late radiotherapy toxicity,” and “single nucleotide polymorphism.” The second phase spans from approximately 2011 to 2017, represented by the keywords “lung cancer histology,” “breast cancer,” “tumor heterogeneity,” “contrast enhanced MRI,” and “F-18 FDG PET” (fludeoxyglucose F18 positron emission tomography). The third phase is after 2018, represented by the keywords “phenotypes,” “big data,” “magnetic resonance spectroscopy,” “renal cell carcinoma,” “stage,” “EGFR mutation,” “temozolomide,” and “artificial intelligence.” 

## Discussion

### Principal Findings

The concept of precision medicine has propelled increased attention toward radiogenomics, a fusion of genomics and radiomics, to achieve personalized treatment, owing to its potential as a noninvasive tool to predict treatment responses. This study analyzed 3237 relevant documents in the field of radiogenomics published between 2005 and 2023 from the WoSCC. The increasing number of annual publications, especially the extremely high growth rate after 2017, indicates how interest in radiogenomics research in the clinical field has been increasing from year to year.

### Current Status of Publications for Countries and Authors

China currently has the highest number of publications in radiogenomics, although the total citation count is lower than that for the United States, with the average citation number per item lower than that of the other top 10 countries. The Netherlands, the United States, and Canada obtained the highest averages per item. It can be concluded that the United States and the Netherlands have performed reasonably well both in terms of the number and quality of published documents, demonstrating their strong influence in the field. Philippe Lambin of the Netherlands ranked first in both number of publications and number of citations, indicating his major contributions to this field.

### Dynamic Publication Trend and Evolutionary Trajectory

The time frame of publications in this relatively new field can be artificially segmented into three phases according to the evolution of hot topics. In the first phase (2005 to 2010), radiogenomics primarily focused on the genetic variation associated with the response to radiation therapy in the field of radiation oncology [12]. Radiation therapy plays a crucial role in tumor treatment, accounting for 50% of all tumor therapies performed worldwide [21]. However, individuals with similar tumors often exhibit significant differences in radiosensitivity, and many patients experience various types of adverse reactions, including radiation-induced lung injury and late radiotherapy toxicity, after radiation therapy [22,23]. To develop precise and personalized treatments that achieve the best efficacy with minimal adverse reactions, researchers have been searching for biomarkers that can predict treatment outcomes. Through analysis of the complete genome using techniques such as genome-wide association analysis, particularly focusing on single-nucleotide polymorphism markers, researchers have identified numerous genomic variation sites associated with the response to radiotherapy [24,25].

In the second phase (2011 to 2017), the concept of radiogenomics expanded. Studies incorporating medical imaging features and biological parameters beyond genomics were also included in radiogenomics studies [26]. It is believed that the features from medical images such as MRI, CT, and PET-CT of lesions are closely related to tumor heterogeneity. Therefore, researchers have extracted the features (including semantic features and texture analysis features) of the tumors and adopted radiomics for a differentiation diagnosis, such as histological subtype identification. Doshi et al [27] found that MRI-based first-order texture metrics can help discriminate between type 1 and type 2 papillary renal cell carcinoma.

From the late second phase onward (ie, 2017 to 2023), the purpose of radiogenomics is not only limited to the prediction of radiotherapy side effects or differential diagnosis but also to analyzing the relationship between gene expression and imaging data. For example, through the analysis of quantitative features of enhanced MRI, Yeh et al [28] found that partial features were correlated with the expression levels of molecules in the Janus kinase-signal transducer and activator of transcription and vascular endothelial growth factor signaling pathways in breast cancer [28].

The distinction between the second and third phases is unclear, with some of the hot topics beginning during the second phase and continuing beyond 2018. In the third phase, the scope of radiogenomics has gradually expanded and become more comprehensive. From the view of raw data, apart from conventional images, some functional imaging techniques such as magnetic resonance spectroscopy have started to be used for radigenomics analysis [29]. Moreover, with the use of a picture archiving and communication system, the storage and re-extraction of medical data are more convenient, which promotes the progress of big data research and improves the credibility of radiogenomics. From the research purposes perspective, more and more therapeutic methods (eg, neoadjuvant therapy, chemoarterial chemoembolization, transcatheter arterial chemoembolization) have been developed and applied in clinical practice. Researchers are beginning to explore the use of radiogenomics to identify patients who may not be sensitive to certain therapies, thereby reducing unnecessary treatment to avoid side effects [30]. From the view of research methods, the studies in the second phase tended to screen for the quantitative features (which were manually extracted in most cases) associated with gene expression status. At present, many studies use machine learning algorithms that are sometimes combined with deep learning algorithms, which can automatically segment lesions to achieve higher predictive performance [27].

### Limitations

Our study has several limitations. First, only research articles and review articles published in English from the WoSCC were included in this analysis, potentially introducing language, publication type, and database biases. Second, this study focused on an in-depth analysis of the dynamic trend and evolutionary trajectory in radiogenomics based on the keywords and references. There are other analyses that could have been considered to better understand the evolution of radiogenomics as a subject, such as more comparative analyses of various factors (ie, authors, countries, keywords, and journals). Third, our results showed that radiogenomics is currently applied mostly in cancer research. Bibliometrics may overlook other topics that are not current research hot spots in the field. For example, keywords related to nononcologic diseases such as mental illness are not included in the tables and figures.

### Conclusion

In conclusion, radiogenomics has attracted substantial attention in recent years. The United States and the Netherlands are the leading countries publishing research in this field, obtaining the highest total citations and average per item, respectively. Before 2010, radiogenomics was mainly used to explore the genetic factors associated with radiotherapy-induced toxicity. Subsequently, the field has evolved to encompass the combination of radiomics and genomics, enabling the prediction of cancer histology, gene mutations, and gene expression status based on the tumor heterogeneity information obtained from medical imaging. More and more researchers tend to be exploring the feasibility of radiogenomics to predict the response of tumors to various treatments such as neoadjuvant chemotherapy. The application of artificial intelligence methods based on big data is emerging as a hot spot research topic in this field at present.

## Appendix

Multimedia Appendix 1 Primary data downloaded as plain text-files from the Web of Science Core Collection (WoSCC) database for bibliometric analysis.

Multimedia Appendix 2 Synonym substitution of keywords.

## Abbreviations

BC: betweenness centrality
CT: computed tomography
EGFR: epidermal growth factor receptor
F-18 FDG PET: fludeoxyglucose F18 positron emission tomography
MRI: magnetic resonance imaging
WoSCC: Web of Science Core Collection
