# Supplementary material for: Research Trends and Evolution in Radiogenomics (2005-2023): Bibliometric Analysis
Source: Interact J Med Res. 2024 Jul 9;13:e51347. doi: 10.2196/51347 (PMC11267093; doi:10.2196/51347)
Supplement: Multimedia Appendix 2 [file ijmr_v13i1e51347_app2.doc]

| label | replace by |
| --- | --- |
| 1p/19q status | 1p/19q |
| artificial intelligence (ai) | artificial intelligence |
| artificial-intelligence | artificial intelligence |
| association | associations |
| biomarker | biomarkers |
| brain metastases | brain metastasis |
| brain tumors | brain tumor |
| brain-tumors | brain tumor |
| breast-cancer | breast cancer |
| combination | complications |
| cancer | carcinoma |
| cancer-patients | cancer patients |
| computer-aided detection | computer-aided diagnosis |
| cell | cells |
| chemoradiation | chemoradiotherapy |
| classifiers | classification |
| colorectal-cancer | coloreCTal cancer |
| computed tomography | CT |
| computed tomography (ct) | CT |
| computed-tomography | CT |
| contrast-enhanced computed tomography | contrast-enhanced CT |
| contrast-enhanced ct | contrast-enhanced CT |
| contrast-enhanced mri | contrast-enhanced MRI |
| ct texture | CT texture analysis |
| ct texture analysis | CT texture analysis |
| ct | CT |
| ct images | CT |
| dce-mri | DCE-MRI |
| diffusion-weighted imaging | DWI |
| diffusion-weighted mri | DWI |
| dna | DNA |
| epidermal growth factor receptor (egfr) | epidermal growth factor receptor |
| egfr | epidermal growth factor receptor |
| egfr mutation | epidermal growth factor receptor mutation |
| egfr mutation status | epidermal growth factor receptor mutation |
| egfr mutations | epidermal growth factor receptor mutation |
| fdg-pet/ct | f-18-fdg pet/ct |
| f-18-fdg pet/ct | f-18-fdg pet/ct |
| fdg pet | f-18-fdg pet |
| fdg pet/ct | f-18-fdg pet/ct |
| fdg-pet | f-18-fdg pet |
| feature-selection | feature selection |
| gastric cancer | gastric-cancer |
| gastrointestinal stromal tumor | gastrointestinal stromal tumors |
| gbm | glioblastoma |
| gene-expression | gene expression |
| gene-expression programs | gene expression |
| glioma | gliomas |
| guideline | guidelines |
| head and neck cancer | head and neck squamous cell carcinoma |
| hepatocellular-carcinoma | hepatocellular carcinoma |
| her2 | human epidermal growth factor receptor 2 |
| high-grade glioma | high-grade gliomas |
| hpv | HPV |
| images | imaging |
| imaging biomarker | imaging biomarkers |
| informatics | information |
| intratumor heterogeneity | intratumoral heterogeneity |
| ki67 | ki-67 |
| kras mutation | kras mutations |
| liver metastases | liver metastasis |
| low-grade glioma | lower-grade gliomas |
| lymph-node metastasis | lymph node metastasis |
| lung-cancer | lung cancer |
| machine learning (machine learning) | machine learning |
| machine-learning | machine learning |
| ml | machine learning |
| magnetic resonance imaging | machine learning |
| magnetic resonance imaging (mri) | MRI |
| magnetic-resonance | MR |
| marker | markers |
| metastases | metastasis |
| mr | MR |
| molecular subgroups | molecular subtypes |
| molecular subtype | molecular subtypes |
| model | models |
| mri | MRI |
| mri features | MRI features |
| multiparametric magnetic resonance imaging | multiparametric MRI |
| multiparametric mri | multiparametric MRI |
| neural-networks | neural network |
| neural-network | neural network |
| non-small cell lung cancer (nsclc) | non-small cell lung cancer |
| nsclc | non-small cell lung cancer |
| non-small-cell lung cancer | non-small cell lung cancer |
| pathway | pathways |
| pattern | patterns |
| pet | f-18-fdg pet |
| pet images | f-18-fdg pet |
| pet/ct | f-18-fdg pet/ct |
| phenotype | phenotypes |
| predict | predictors |
| prediction | predictors |
| prediction model | predictive models |
| predictive model | predictive models |
| predictor | predictors |
| prognostic-factor | prognostic-factors |
| pulmonary nodule | pulmonary nodules |
| prostate-cancer | prostate cancer |
| radiation therapy | radiation-therapy |
| radiomic | radiomics |
| radiomic features | radiomics features |
| rectal cancer | rectal-cancer |
| renal-cell carcinoma | renal cell carcinoma |
| renal-cell-carcinoma | renal cell carcinoma |
| signature | signatures |
| single nucleotide polymorphisms | single-nucleotide polymorphisms |
| neoadjuvant chemoradiotherapy | neoadjuvant chemotherapy |
| survival | survival analysis |
| textural features | texture features |
| tumor | tumors |
| ultrasonography | ultrasound |
| gene expression programs | gene expression |
| ai | artificial intelligence |
